# Leaky Wave Generation Through a Phased-Patch Array

**DOI:** 10.3390/s25092754

**Published:** 2025-04-26

**Authors:** Alessandro Calcaterra, Patrizio Simeoni, Marco Donald Migliore, Fabrizio Frezza

**Affiliations:** 1 Department of Information Engineering, Electronics and Telecommunications, La Sapienza University of Rome, 00184 Rome, Italy; 2 Department of Electronics Engineering and Communications, South East Technological University (SETU), Carlow Campus, R93 V960 Carlow, Ireland; 3 Dipartimento di Ingegneria Elettrica e dell’Informazione (DIEI) “Maurizio Scarano”, University of Cassino and Southern Lazio, 03043 Cassino, Italy

**Keywords:** leaky waves, leaky-wave antennas, inhomogeneous waves

## Abstract

For this article, we approximated the field of a leaky-wave antenna (LWA) with the field produced by a uniform linear array (ULA). This article aims to provide an initial framework for applications where the generation of an inhomogeneous wave is wished, but, at the same time, a flexibility is required that is difficult to meet with the conventional LWA design. In particular, two different configurations were considered, one with a simple Menzel antenna operating at 12 GHz, and one, relevant for practical applications, with an antenna operating at 2.4 GHz. This study aimed, in both cases, to highlight the distance at which the field produced by the phased array with the chosen sampling method can approximate effectively the one produced by a leaky-wave antenna and to verify whether this could cause issues for the targeted application.

## 1. Introduction

It has been demonstrated that inhomogeneous waves can enable deeper penetration in lossy media. Such waves can often be approximated by the field produced by a leaky-wave antenna. However, in specific applications—such as hyperthermia treatment, where the radiating environment (i.e., biological tissues) varies from patient to patient—generating a precise field configuration requires tuning that is challenging to achieve with classical leaky-wave antennas, particularly under deep penetration constraints [1]. In such cases, an alternative approach that maintains the desired field characteristics while offering greater flexibility may yield better results. This can be achieved through the use of a phased array. Leaky waves are the only inhomogeneous waves capable of delivering power in a lossless medium, as they are not bounded to a surface, as happens with surface waves, plasmonic polaritons, or others [2]. As with surface waves, on the other hand, leaky waves are waves that appear only in the presence of a discontinuity or an asymmetry, in general [3]. Leaky-wave antennas are well-known artificial structures used to generate near-field radiation that closely approximates a leaky wave, and they are traditionally employed to produce a leaky-wave field. In this article, we aim to replace the leaky-wave antenna with a phased array. As is well known in the literature, the field produced by a continuous aperture antenna can be approximated by that of an array by sampling the currents on the antenna aperture [4]. As stated in [4], the accuracy of this approximation increases as the distance between the array elements decreases. In the limit, the two fields become identical, though this is clearly impractical. When the spacing between elements is large, the fields between the two antennas may not match well and direct sampling may not provide good results. Solutions to this problem have been proposed in the literature, including root-matching methods and perturbation techniques [4,5], as well as more recent integrated sampling methods [4,6]. The need for these methods highlights the complexity of achieving a sufficiently accurate approximation of the field produced by an antenna array compared to that of a continuous antenna. For this reason, this paper investigated whether, in the case considered, simple sampling at a distance of λ/2 is sufficient for the application proposed in [7], by first comparing the far fields and then the near fields produced by the two antennas. This study represents an initial step in that direction. In the literature, many LWAs structures have been proposed, to address aspects such as miniaturisation, directivity, full scanning, and stop-band suppression [8], but they were not strictly relevant to the goal of this article. For this work, therefore, we chose a simple LWA—the Menzel antenna—due to its specific attenuation and phase vector properties [9]. A practical way to realise a leaky-wave antenna (LWA) is to consider a travelling-wave structure with several asymmetries properly designed to implement a given value for the attenuation and propagation constants [10,11,12].

**Figure 1:**
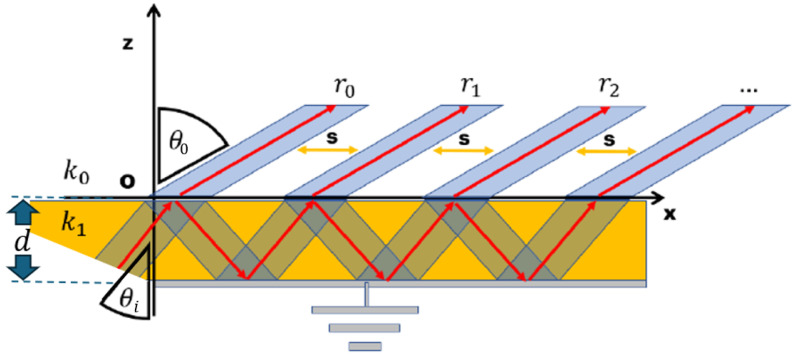
Pictorial representation of leaky radiation principle.

In the literature, a well-known interpretation of the leaky-wave radiation phenomenon also explains the presence of a shadow cone where the field behaves improperly, as shown in Figure 1 [11]:

Inside the dielectric, as is well known from the electromagnetic theory [13,14,15], whenever the field reaches the dielectric–air interface, it is partially reflected and transmitted. The reflection and transmission coefficients, also known as Fresnel coefficients, depend on the angle of incidence, the electromagnetic characteristics of the two media involved, and on the polarization (TE or TM) of the considered field [13,14,15].

What is important is that at each incidence a leakage occurs, generating a propagating field in the air that moves away from the interface. The ratio of this field with respect to the impinging one is, by definition, the transmission coefficient [13,14,15]. The reflected field, on the other hand, keeps propagating inside the waveguide. Again, the remaining power will only be a portion of the initial impinging field, due to the leakage.

This leakage rate per meter is the attenuation constant that, due to the conservation of the tangential component of the electric field at the interface, is present in the field radiated in the air.

It is well known that the behaviour of the field inside the guide is frequency-dependent. In fact, starting from the cut-off frequency at which the field does not travel inside the dielectric (the stop-band region), by increasing the frequency the propagation vector points more and more towards the propagation direction shown in Figure 1 (i.e., x direction). Again, this is a good explanation of how a leaky-wave antenna operates by varying the frequency: the higher the frequency, the more the field is radiated towards endfire.

To analyse the numerous types of existing leaky-wave antennas was beyond the scope of this paper. We only considered a uniform monodimensional LWA, where the cut-off of the guided mode of interest coincides with the stop band.

The analysis and design of a given LWA requires the derivation of its dispersion diagram and the calculation of the attenuation and propagation constants related to the geometrical and electromagnetic characteristics of the waveguide and its asymmetries. Finally, the attenuation and propagation constant are related to the far-field properties.

The following approximate relationships are present in the literature, valid for α<<β, where α is the absolute value of the attenuation vector and β is the absolute value of the phase vector:

(1) $$\theta_{0}≅sin^{-1}\left(\beta_{z}/k_{0}\right)$$

(2) $$\theta_{3dB}≅\frac{0.91}{\left(L/\lambda_{0}\right)cos\theta_{0}}\propto \frac{\alpha }{k_{0}}$$

In the equations illustrated above, $\theta_{0}$ is the angle between the normal and the antenna aperture and the radiated beam, $\theta_{3dB}$ represents the beamwidth, λ is the wavelength, $\beta_{z}$ is the *z* component of the phase vector, and $k_{0}$ is the wave number in vacuum. These relations imply that the pointing angle is decided by the value of the propagation constant β, while the beamwidth is decided by the attenuation constant α. The intuitive explanation of the frequency behaviour has been provided above. As far as the relationship between the attenuation constant and the beamwidth is concerned, it is sufficient to note that a larger attenuation results in an earlier decay of the electromagnetic field and so in a shorter electrical length and, as is known, a wider beam.

### Phased Array

The properties of a phased array are well known and will not be analysed in detail here. The interested reader may refer to [4,16].

It is worth recalling here that the active electronically scanning array (AESA) can produce a beam that scans the space by simply modifying the phase of each element. In particular, the scanning is achieved by satisfying a condition on the phase shift to allow constructive interference between the fields produced by the radiating elements along the direction of interest.

For active linear uniform phased arrays, this condition is

(3) $$\Delta \phi =kd[sin\left(\theta \right)-sin\left(\theta_{0}\right)]$$

where *k* is the wave number and *d* is the fixed distance between elements. The amplitude of the excitation phasor of each radiating element impacts the pattern shape. Typically, this property is used to reduce side lobes by applying proper tapering [16], i.e., reducing the amplitude of elements close to the edges. Intuitively, this happens because by reducing the currents on the edges the antenna results are electrically smaller, leading to a wider main beam and lower side lobes.

So, to achieve the desired pattern, i.e., to impose the desired set of coefficients for the radiating elements, it is necessary to modulate the amplitude and phase of each element. A typical solution that allows for achieving this employs a Digital Variable Attenuator to control the amplitudes and Digital Phase Shifters to modify the phases. These are components with a given number of bits (that ranges typically from 6 to 8) that introduce either an attenuation or a phase shift. Introducing a phase shift between radiating elements assures the beam pointing for a specific frequency. This means that for higher or lower frequencies, the relation (3) is no longer valid, resulting in a beam that squints around the direction of interest. This topic will be further developed in the Results section of this paper, where the behaviour of the phased array is compared to that of the LWA.

This article aims to establish an alternative and improved design of the antenna proposed in [7], which utilizes inhomogeneous waves for hyperthermia applications, as they may enable deeper tissue penetration. In this study, we focused on a numerically feasible scenario by considering an antenna operating at 12 GHz as a feasibility study. Then, we explored its application to hyperthermia by designing a prototype operating at 2.4 GHz, paving the way for more advanced antenna designs and experimental measurements.

## 2. Materials and Methods

All the simulations were performed with commercial electromagnetic software that implements finite integration techniques (FITs) in Time Domain [17,18]. The choice of the FIT Solver for all the structures considered here was due to its granting the same simulation conditions for the antennas. For the LWAs, the correct operating mode was excited through a waveguide port. For the array, coaxial connectors were designed to provide a 50 Ohm feeding line to the patch antennas. Finally, all the metallizations were perfect electric conductors (PECs), and the dielectrics (that were the same for both the LWAs and the patch arrays) were loss-free. These latter choices were made to improve the simulation time, and they do not represent a restriction of generality with respect to the paper’s purposes. More specifically, the antenna has been designed and simulated by using the CST Microwave Studio Software [19] licensed to the DIET Department of “La Sapienza” University of Rome, and all figures shown in the paper have been obtained either by producing them with such a software, or re-designing them, eventually using MATLAB [20], with the addition of custom information, aimed at providing additional details which were not available in the original figures. Also, Microsoft Powerpoint [21] has been used to add additional information, e.g., geometrical dimensions, to the antenna designs produced.

### 2.1. The Menzel Antenna Operating at 12 GHz

**Figure 2:**
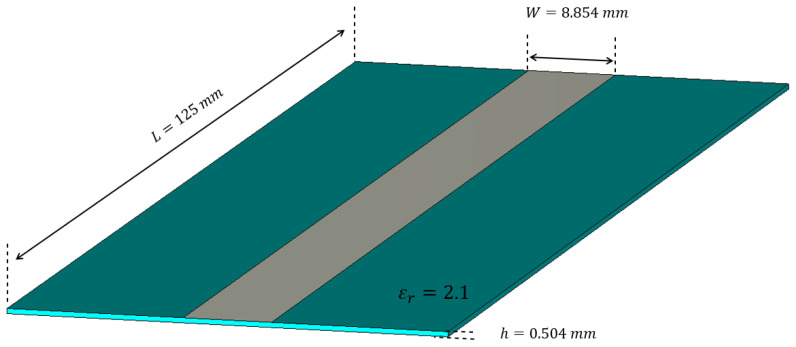
Design of the Menzel antenna.

The LWA chosen for this analysis is based on the Menzel antenna, already considered in [22,23]. Its geometrical characteristics are recalled in Figure 2. In this case, a waveguide port is placed on the antenna termination to absorb all the non-radiated field.

**Figure 3:**
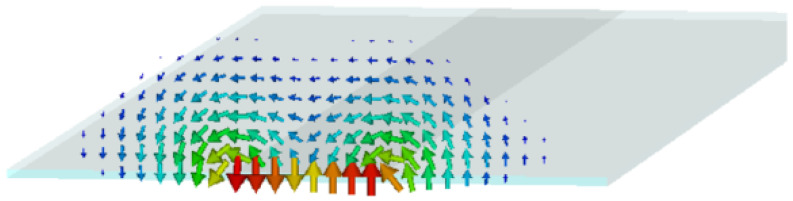
Radiating mode of the Menzel antenna.

This antenna radiates in its odd higher-order modes [22,23]; we chose here to excite the first higher-order odd mode, simulated and plotted in Figure 3.

In a simulation environment capable of separately analysing the modes of a waveguide it is possible to selectively excite the mode of interest.

At the prototyping stage, it is necessary to suppress the fundamental (Q-TEM) and the first even higher-order mode; however, as the scope of this paper was to highlight and demonstrate the similarities between a phased array and a leaky-wave antenna in a simulation environment we did not consider a more feasible model for the LWA, such as the one presented in [7]; we focused, instead, on the electromagnetic radiation of interest.

**Figure 4:**
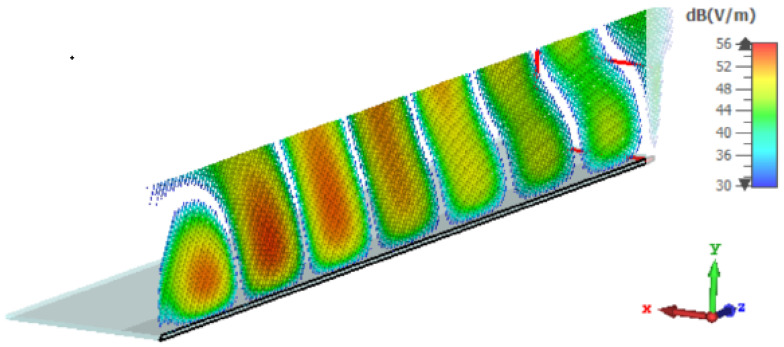
Near field of the Menzel antenna.

**Figure 5:**
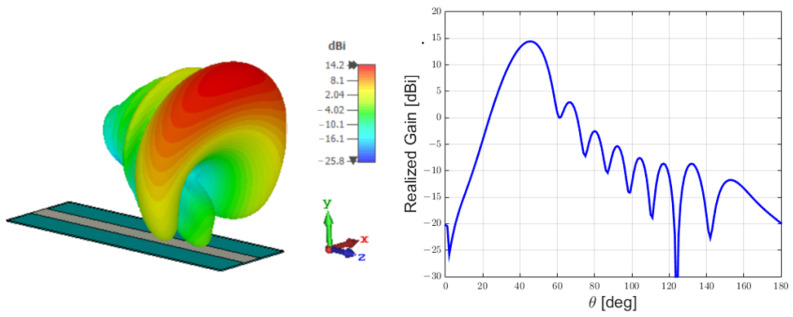
Far field of the Menzel antenna.

In Figure 4 and Figure 5, respectively, the LWA near field and far field are shown:

### 2.2. The Sampling Methodology

To sample the current on the aperture, we had to choose the elementary radiating elements and their locations, i.e., the spatial sampling. In regard to the radiating element, a simple patch antenna [24,25] was chosen, due to its similarity to the leaky-wave antenna considered.

**Figure 6:**
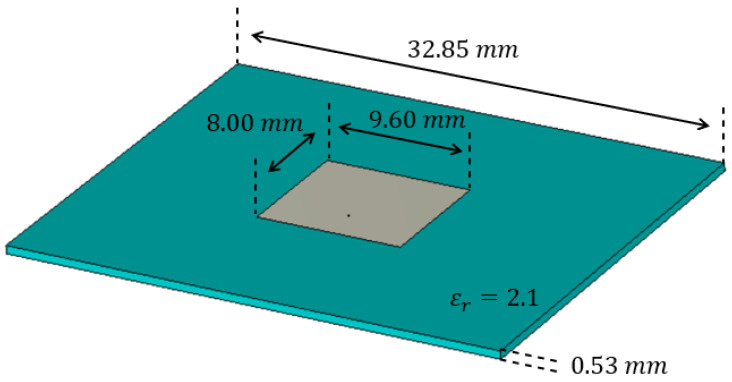
Patch antenna used as element of the array.

**Figure 7:**
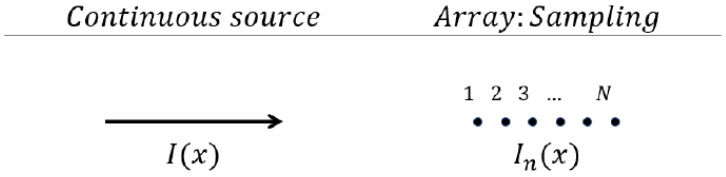
Schematic representation of a continuous current source (on the **left**) and of a discrete current source obtained by sampling the continuous source (on the **right**).

Figure 6 shows the patch antenna that was designed as an element of the array: even if it was not necessary, the same substrate of the LWA was used:

When in the array, the patch antenna had to be placed so that the field was polarized in the same direction as the one radiated by the leaky-wave antenna; see Figure 7. To determine the exact number of patches in the array, a straightforward observation could be made: in order to radiate the same field, it was necessary that the two structures had the same currents on the same electrical area. The simplest way to assure this for the two antennas considered was to make them the same geometrical length.

Placing the patches too close together would have resulted in a poor active reflection coefficient, due to the mutual coupling. On the other hand, it was not advisable to place them too far apart, since grating lobes might occur and, furthermore, a coarse sampling would have resulted in a poor representation of the field emitted by the LWA.

For these reasons, it was decided to keep λ/2 spacing between the patches: this was the maximum distance that allowed us to avoid grating the lobes in the visible region when scanning up to endfire. Given the geometry considered, the array that we designed consisted of 9 radiating elements (patches) placed at a constant distance.

To finalize the array design, it was essential to define the amplitudes and phases of the patch elements. Regarding the phases, we began by setting the LWA to radiate at a $\theta^{LWA}=45^{\circ }$ angle; then, the phases for the array were set to values equal to the ones required by a phased array to point the beam in the same direction as the LWA.

**Figure 8:**
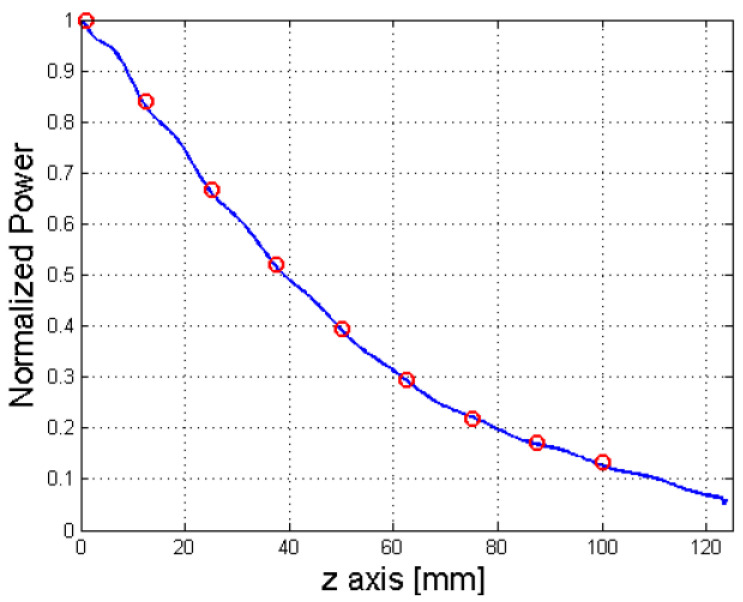
Sampling of the power inside the dielectric of the LWA.

For the amplitudes, the normalised power in the dielectric of the LWA was simulated along the longitudinal direction. Then, it was sampled at each position in which a radiating element was present in the phased array along the symmetry axis. Figure 8 illustrates the resulting power distribution along the longitudinal direction of the patch antenna.

**Figure 9:**
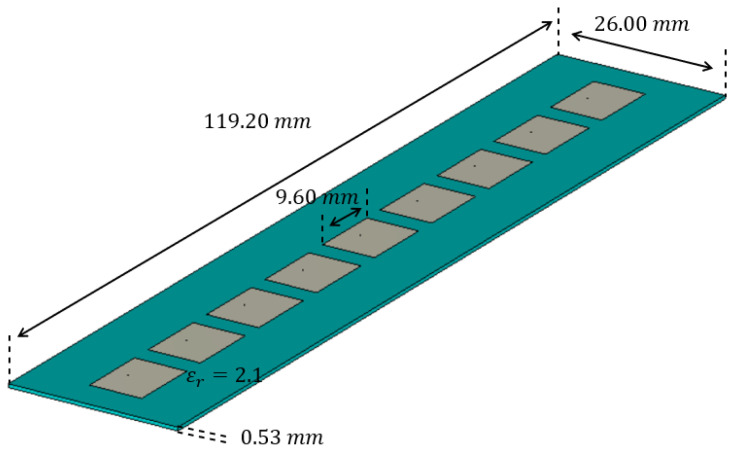
Layout of the phased array constituted by the patch antennas chosen.

**Figure 10:**
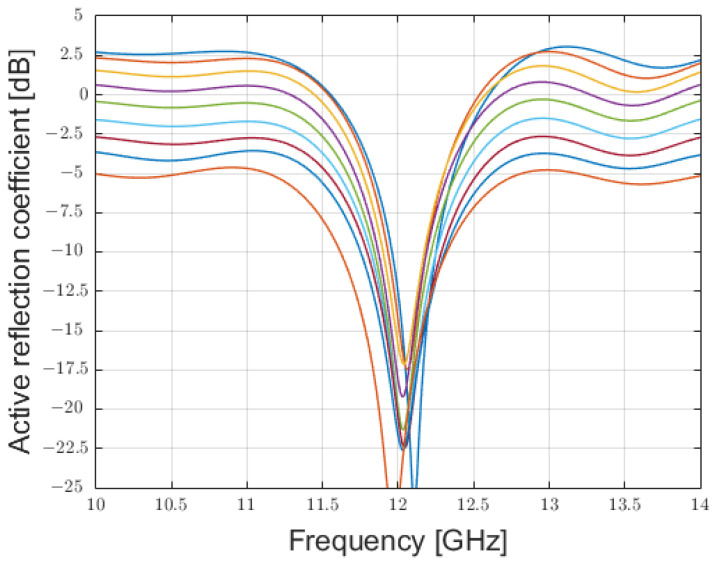
Active reflection coefficients for the 9 patch-antenna elements composing the phased array excited with the coefficients reported in Table 1.

**Figure 11:**
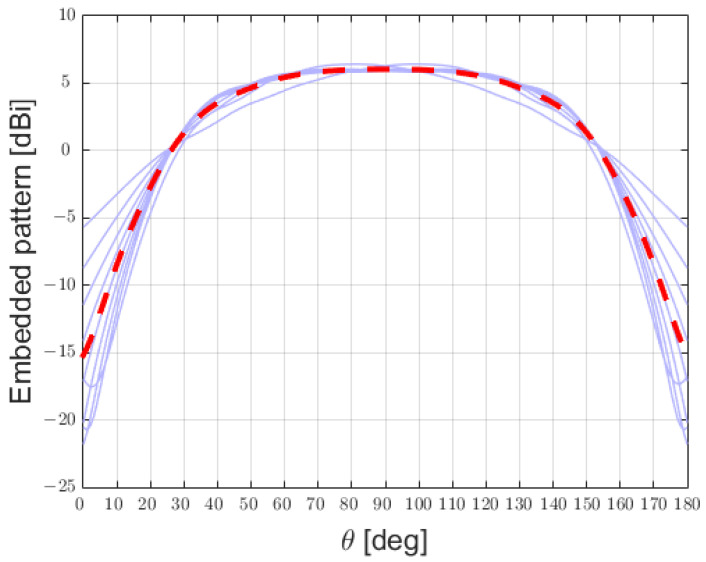
Embedded patterns of the 9 patch-antenna elements composing the phased array (in purple) and the average embedded pattern (in dashed red).

The phasors used for the 9 elements are represented in Table 1:

With these assumptions, the patch array of Figure 9 was realised:

The performances of the array obtained could be rapidly assessed by evaluating the active reflection coefficient of each element (Figure 10) and by the average embedded element pattern (Figure 11):

### 2.3. Analysis of an Antenna Operating at 2.4 GHz

**Figure 12:**
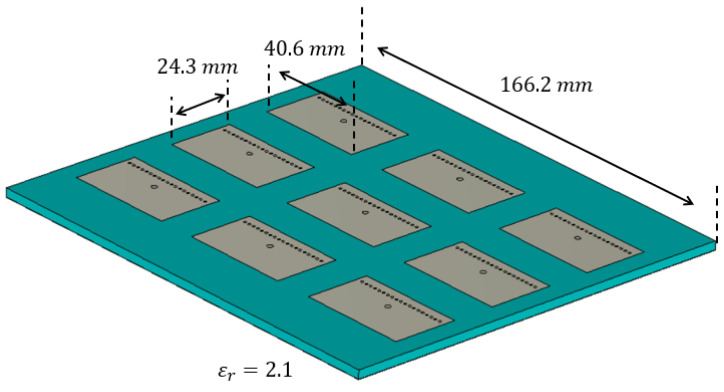
Design of a compact uniform LWA realised employing a phased array.

We then focused on a practical application, i.e., an LWA for hyperthermia treatment operating at 2.4 GHz. It is well known that in LWAs there may be an excess of power at the end of the antenna; this problem becomes more relevant when trying to minimize antenna dimensions. In the literature, different methods have been employed, therefore, to try re-using the power that is available at the end of the LWA, in order to improve the efficiency [7,26,27,28]. Where frequency scanning is required, a design such as the one implemented in [27] could be considered, while in applications like the one in [7], where a specific incidence angle alongside a wave that well approximates a single inhomogeneous wave is desired, the alternative approach proposed here can also be explored. For a near-field application, it is pivotal to verify the distance from the antenna at which the phased array well approximates the leaky-wave antenna. To demonstrate whether an acceptable field can be achieved at a short distance from the array, we took into consideration the design proposed in [7], as a reference design, and we eliminated the reliance on the Wilkinson splitter, which has the downside of requiring an additional PCB, by designing the array shown in Figure 12:

## 3. Results

### 3.1. Comparison Between the Antennas Operating at 12 GHz

**Figure 13:**
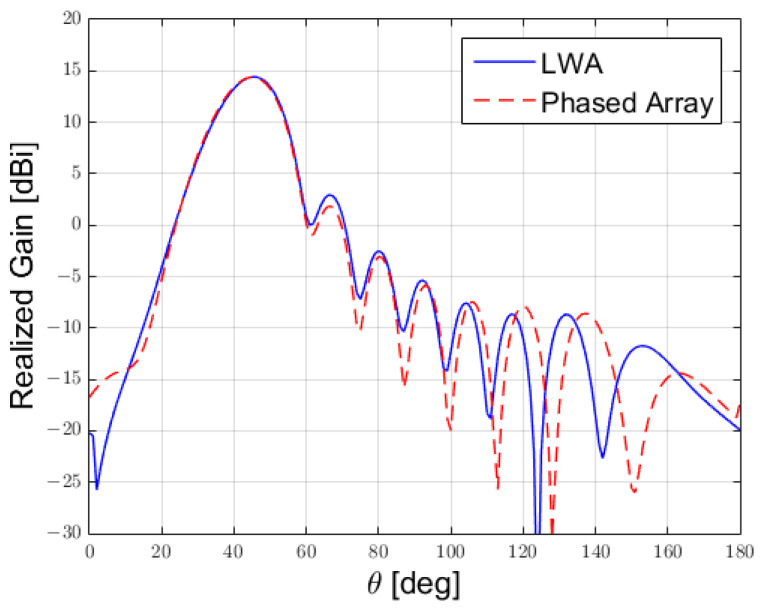
Comparison between the far field produced by the LWA versus the one produced by the phased array.

We first compared the two antennas in terms of the generated far field, because the far field was obtained via an integral transformation of the near-field radiation, resulting in a preliminary estimator of the similarity among the two structures; see Figure 13. Also, this provided important information related to the radiating properties of the two antennas.

We then examined the near field, to identify the maximum vertical distance from the antenna where the array still produced a field that satisfactorily approximated the one generated by the Menzel antenna.

After having verified the similarity in the far field, before focusing on the near field, it is worth making some observations to extend the comparison between these two structures to the frequency-scan behaviour.

It is well known that the LWA radiation mechanism is given by the radiation losses provoked into a guiding structure. So, it is clear that the propagating mode inside the considered lossy waveguide is of chief importance as far as the radiation of the LWA is concerned. If this mode is in cut-off, there will be no radiation at all. By scanning the frequency, the mode will start propagating inside the structure (i.e., its wave number component along the direction of propagation passes from predominantly imaginary to predominantly real), and the LWA will start radiating near broadside. Again, increasing the frequency, the LWA will scan the beam up to endfire.

For the reasons just stated, for a one-dimensional LWA it is impossible to radiate at broadside, since it would correspond to the radiation when the mode inside the guide is in cut-off. This issue, known as stop-band [11], can be overcome in several ways that will not be considered here. The interested reader may refer to the relevant literature, e.g., Ref. [11].

For a phased array, the situation is quite different. In this case, a propagating mode inside the structure is not present, and, to enforce a radiation angle for the beam, a specific phase relation between the radiating elements has been super-imposed here. It is then interesting to analyse what happens when the frequency varies, in particular focusing on the resulting spatial geometrical scanning of the radiated beam. In this case, the direction of scanning can be computed manipulating Equation (3), which we write here for the sake of clarity, it is:

(4) $$\Delta \phi =kd\left[sin\left(\theta \right)-sin\left(\theta_{0}\right)\right]=kdsin\left(\theta \right)-\beta$$

then it follows:

(5) $$\theta_{0}=arcsin\left(\frac{\beta }{kd}\right)=arcsin\left(\frac{\beta }{2\pi fd/c}\right)$$

where Δϕ is the total phase between the adjacent antenna elements, while $\beta =kd\sin(\theta_{0})$ represents the progressive phase shift between the elements of the array [4]. It follows that if the amplitude of β is chosen so that the antenna radiates at a $\theta_{0}=45^{\circ }$ angle for a frequency f=12 GHz then as the frequency increases the radiated beam tends to point to broadside. If the frequency decreases, the beam is directed towards endfire. This behaviour is the opposite with respect to an LWA, but it is very interesting to note that in this case there is also a stop-band behaviour at broadside as, from the relation above, it is clear that to achieve a $\theta_{0}$ equal to 0, the operating frequency needs to tend to infinity. To reach endfire, instead, it is sufficient to reach the frequency, as follows:

(6) $$f=\frac{\beta }{2\pi \sin\theta_{0}d/c}$$

**Figure 14:**
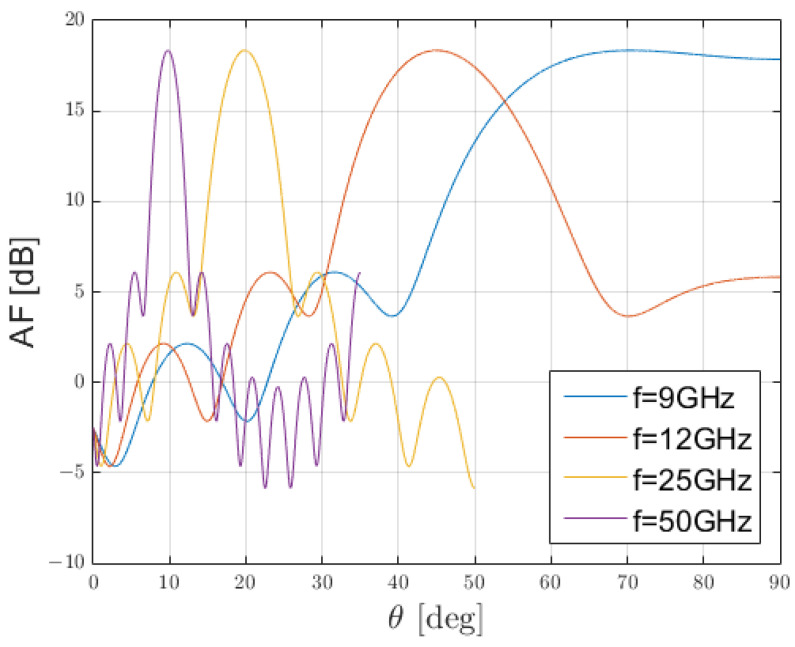
Array factor (AF) of a linear equi-spaced phased array with fixed phase-relation at different frequencies.

To assess this behaviour, let us consider Figure 14, where the array factor of a linear equi-spaced array with a phased coefficient, so as to scan at $45^{\circ }$ at 12 GHz, has been considered at different frequencies. Here, the squinting behaviour is exaggerated to show how, given a set of coefficients to scan at a given angle, a stop-band behaviour is experienced at broadside.

In Figure 14, only the array factor is shown, for three main reasons: firstly, it would not be feasible to realise a radiating element that could cover such a bandwidth. This is even more true for patches that typically cover fractional bandwidths of 1–5% when in standard configuration [29,30]. Secondly, this phenomenon is due to the relative amplitude and phase of the electromagnetic field for the elements in a given lattice. All these characteristics, according to the elementary theory of array, belong to the array factor and not to the element factor that is, or at least should be, common to all radiating elements [16].

After having compared the far fields, we then analysed the differences in the near field, and, given the difference in behaviour in the case of the frequency scan, we concentrated on the situation in which both antennas pointed at a $45^{\circ }$ angle, as in this case the two antennas operated at the same frequency, by design choice.

**Figure 15:**
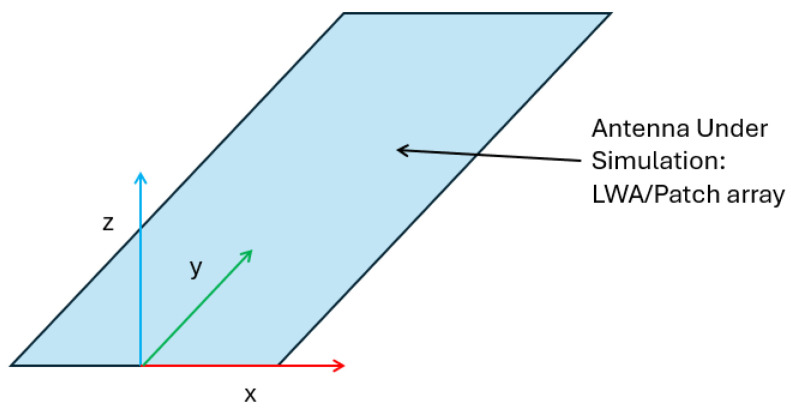
Reference system used for the comparison.

**Figure 16:**
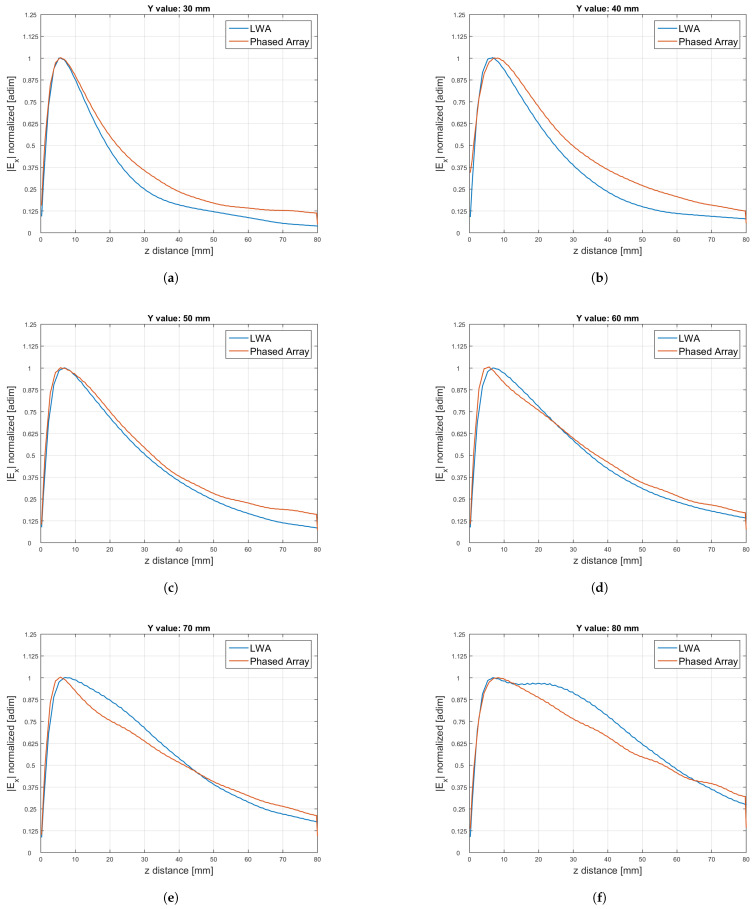
Comparison between the amplitude $|E_{x}|$ of the electric field normalised by its maximum for the LWA and the phased array along the *z* axis when x=0 and for different constant values of the *y* coordinate: (**a**) (x,y)=(0,30) mm; (**b**) (x,y)=(0,40) mm; (**c**) (x,y)=(0,50) mm; (**d**) (x,y)=(0,60) mm; (**e**) (x,y)=(0,70) mm; (**f**) (x,y)=(0,80) mm.

To make this evaluation, referring to the reference system shown in Figure 15, we firstly compared the amplitude of the electric field $|E_{x}\left(0,y_{i},z\right)|$ as a function of *z*, sampling it at a different position, $y_{i}$, where the subscript *i* denoted a distinct sampling point along the *y* axis:

Our results are shown in Figure 16.

**Figure 17:**
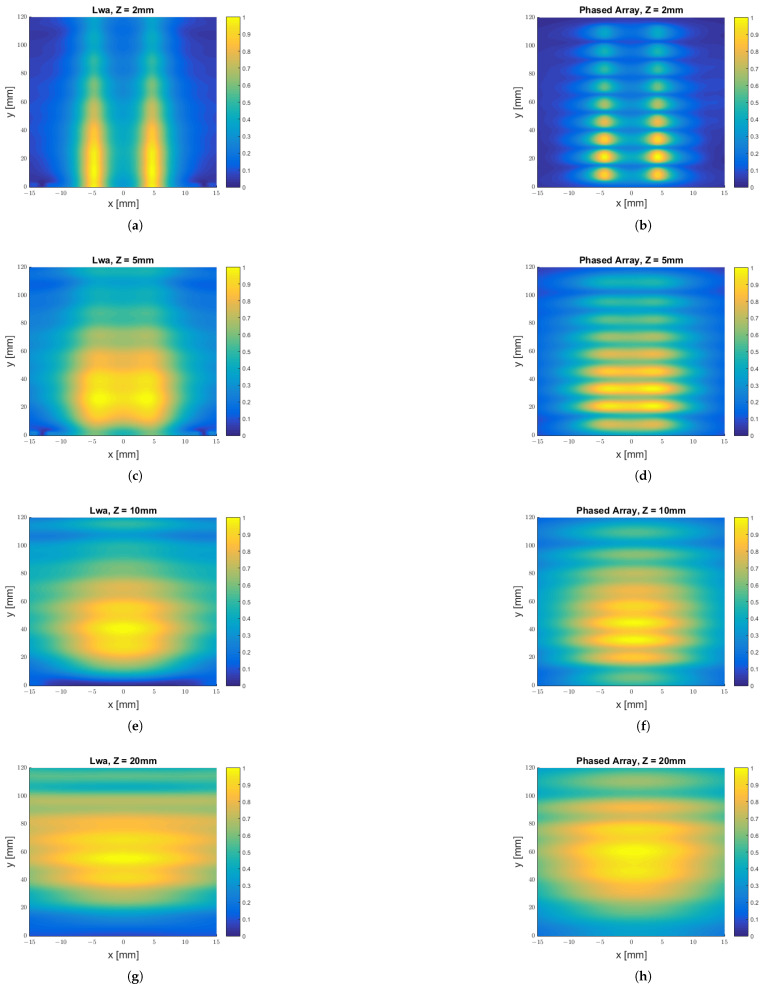
$|E_{x}|$ field component sampled at distinct planes $z=z_{i}$ for the LWA, on the left, and the phased array (PA), on the right: (**a**) $|E_{x}^{LWA}|$ for z=2 mm; (**b**) $|E_{x}^{PA}|$ for z=2 mm; (**c**) $|E_{x}^{LWA}|$ for z=5 mm; (**d**) $|E_{x}^{PA}|$ for z=5 mm; (**e**) $|E_{x}^{LWA}|$ for z=10 mm; (**f**) $|E_{x}^{PA}|$ for z=10 mm; (**g**) $|E_{x}^{LWA}|$ for z=20 mm; (**h**) $|E_{x}^{PA}|$ for z=20 mm.

After this evaluation, we compared the field on planes for different constant values of the *z* coordinate, i.e., we plotted $|E_{x}\left(x,y,z_{i}\right)|$, choosing different values, $z_{i}$, along the *z* axis. Our results are visible in Figure 17.

**Figure 18:**
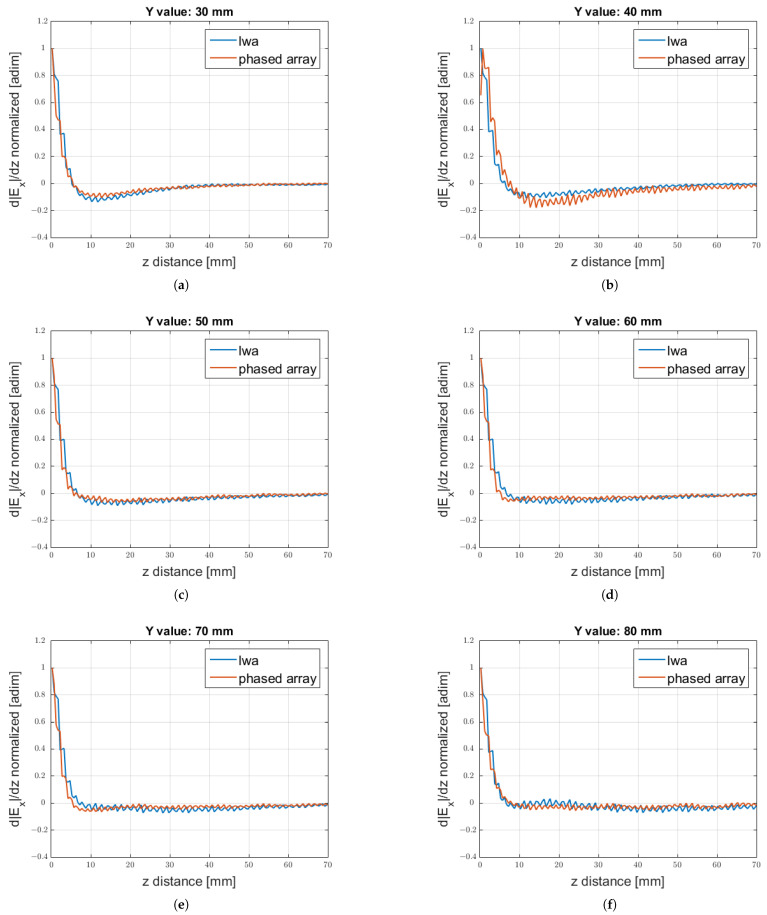
Comparison of the derivative of the $|E_{x}|$ component with respect to y along *z* for different constant values chosen along the *y* axis. Values: (**a**) (x,y)=(0,30) mm; (**b**) (x,y)=(0,40) mm; (**c**) (x,y)=(0,50) mm; (**d**) (x,y)=(0,60) mm; (**e**) (x,y)=(0,70) mm; (**f**) (x,y)=(0,80) mm.

The calculation of the amplitude of the field normalised by its maximum value was a useful estimator for evaluating the behaviour of the two structures without taking into account the different power radiated, i.e., without considering the efficiency of the antennas. Another interesting parameter that allowed us to obtain a deep understanding of the variation of the field was the derivative of such a field, as shown in Figure 18.

**Figure 19:**
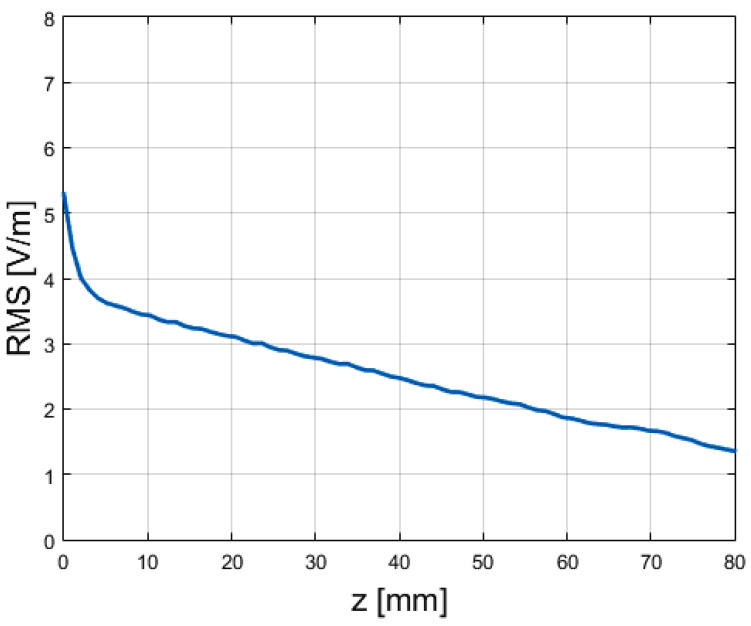
Root-mean square (RMS) error for the amplitude of the electric field between the LWA and the phased array, evaluated as specified by Equation (7).

To make a quantitative evaluation of the similarity, we computed the root mean square (RMS) value of the error, visible in Figure 19. For every point in the *z* direction (i.e., the longitudinal direction for the antenna), we evaluated the RMS error on the field values over the (x,y) plane.

The applied formula was

(7) $$RMS_{err}\left(z\right)=\frac{1}{NM}\sqrt{\sum_{n=1}^{N}\sum_{m=1}^{M}{\left(|E_{x}^{LWA}\left(x_{i},y_{j},z\right)\left|-\right|E_{x}^{pa}\left(x_{i},y_{j},z\right)|\right)}^{2}}$$

where *N*, *M* were the numbers of samples on, respectively, the *x* and *y* axis.

It can be seen that the error rapidly increased as the distances along *z* decreased, where the different radiation mechanisms (continue vs. sampled) became more evident.

### 3.2. Comparison Between the Antennas Operating at 2.4 GHz

**Figure 20:**
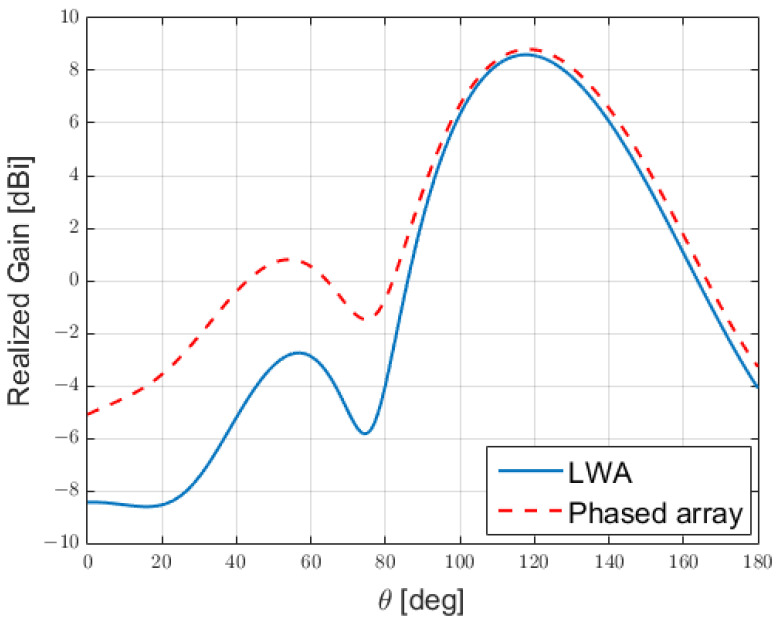
Gain produced by the phased array of Figure 12 compared to the one produced by the LWA of [7].

A procedure, analogous to the one just illustrated, was then applied to the LWA proposed in [7] for hyperthermia treatment, in comparison with the array of Figure 12. The comparison between the far field produced (an indication of the correct sampling), is demonstrated in Figure 20, from which one can easily verify that this early prototype suffered from a large backlobe, due to the limited number of patches employed to reduce the coupling between the array elements, while it produced a main lobe that well resembled the one produced by the antenna in [7].

**Figure 21:**
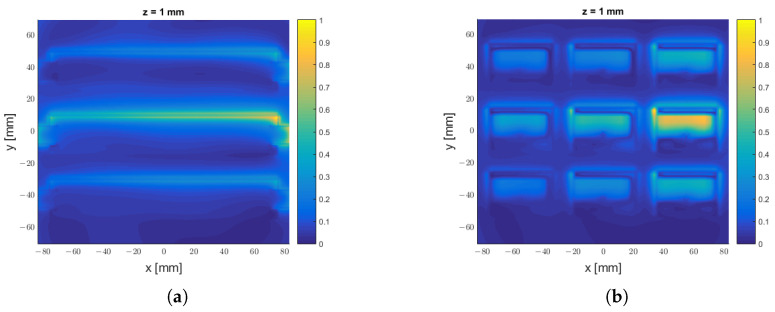

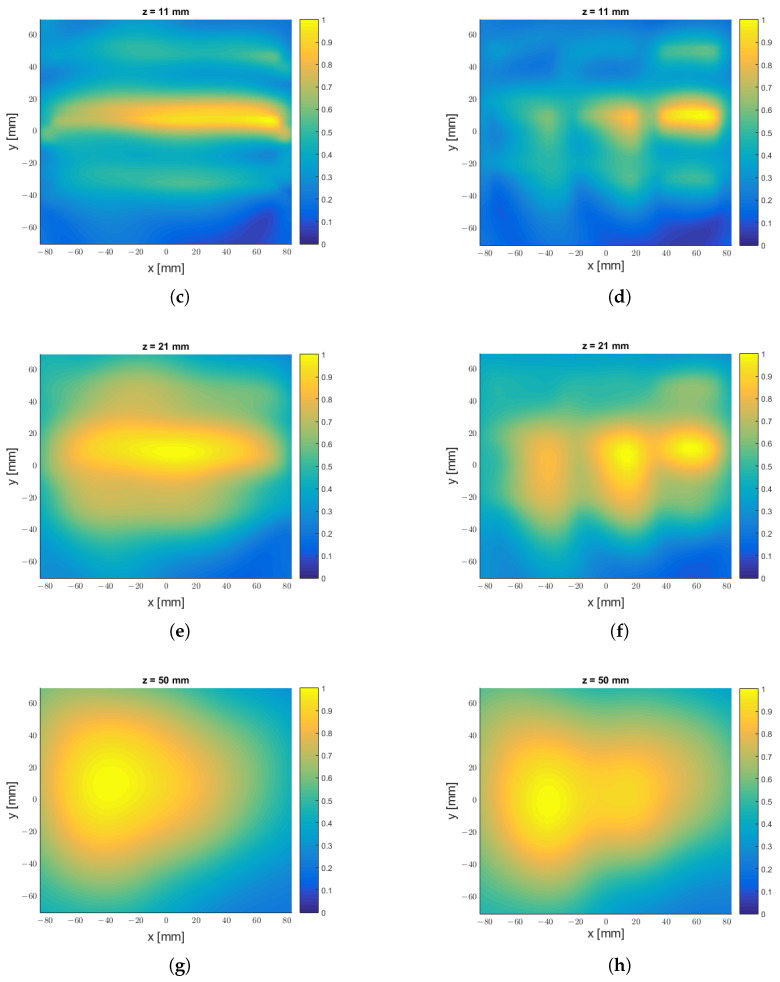
Comparison between the amplitude $|E_{x}|$ of the electric field normalised by its maximum for the LWA in [7] and the phased array designed here along the z=k planes, where z=0 represents the plane at the antenna aperture: (**a**) LWA field at z=1 mm; (**b**) phased-array antenna field at z=1 mm; (**c**) LWA field z=11 mm; (**d**) phased-array antenna field at z=11 mm; (**e**) LWA field z=21 mm; (**f**) phased-array antenna field at z=21 mm; (**g**) LWA field z=50 mm; (**h**) phased-array antenna field at z=50 mm.

Moving to the near-field analysis, we considered the differences in the field produced by the two antennas at different distances from the aperture; see Figure 21. The antenna in [7] started approximating the behaviour of a uniform LWA (a full-size Menzel antenna having the same frequency) at a distance of z=21 mm from the aperture: we observed a similar behaviour for the phased array, although the discretisation of the field caused by the presence of the patches remained noticeable. The approximation to the uniform LWA was instead very good for both antennas at a distance of z=50 mm from the aperture.

## 4. Discussion

In this paper, we have considered the possibility of sampling a continuous distribution of current to achieve a discrete representation of the same field. We chose a leaky-wave antenna to be the first element of the comparison, due to the continuous current that flows on its metallization, and we compared its field with the one produced by a patch-antenna array. The coefficients that excited this array were chosen to be equal in amplitude and phase with the field samples taken from the LWA radiating at a 45-degree angle and at a frequency of 12 GHz, properly chosen to represent the total field. We used four different estimators, one performed in far field and three in near field, to evaluate the agreement of the two structures, also considering the RMS error as the “quality factor” to evaluate the goodness of the process. Depending on the degree of the acceptable agreement, this study has shown that it is possible, within certain limits, to consider an equivalence between the field radiated by a continuous current source and a discrete one.

After this preliminary analysis, we moved to a more practical scenario, where we tried to verify the suitability of the approach taken, by designing an array that mimicked the behaviour of an LWA design recently proposed for hyperthermia treatment.

In this paper, we made use of phase shifters, but other alternatives could be explored in future research. An interesting one would be the application of true time delays, which introduce a delay constant in the frequency. In this way, the squint phenomenon in the frequency does not occur.

Also, it should be noted that since phase shifters have a minimum phase step that can be introduced, there is a quantization error in the phase that can be applied. This is also true for variable attenuators, which cannot introduce any desired attenuation, but this is limited to a fixed step. These technical aspects were not developed in this paper, but a more comprehensive study taking into account these non-idealities will be conducted.

This study also demonstrated that with the approach taken it is possible to mimic the behaviour of a leaky-wave antenna, using only a patch array for a specific frequency. Additionally, the stop band was reproduced. However, while frequency-scanning behaviour can also be achieved with the patch array, it differs significantly from that of a leaky-wave antenna, both in terms of direction (which shifts toward broadside as frequency increases) and frequency range (which is much larger). Future research will need to explore alternative approaches to replicating this additional feature, if it is needed by the considered application.

## 5. Conclusions

This research demonstrates the feasibility of generating a field using a patch array that emulates the behaviour of a leaky-wave antenna (LWA). This finding allowed us to also propose an alternative approach to antenna design for hyperthermia treatment, with a prototype that produces a field that can be approximated with an inhomogeneous wave using a small phased-array antenna. The phased array obtained through the sampling method demonstrated a few challenges, especially in the application at 2.4 GHz, where we needed to control the coupling between the patches, which partially impacted the quality of the approximation to the LWA. This technique also demonstrated advantages, such as enhanced flexibility, that may allow easier adaptation for the amplitudes of the phase and attenuation vectors to specific tissues, and the elimination of both the need to re-use power at the end of the LWA to enhance antenna efficiency and the need to suppress unwanted modes. These advantages would come at the cost of a more challenging implementation, as an array is inherently more complex than an LWA structure. Additionally, our study highlighted significant differences in the scanning behaviour of the leaky-wave and phased-array antennas. Further research is required, to determine whether this aspect of an LWA can also be replicated with a patch array. Future research may focus on approaching the open challenges highlighted in this paper, to improve the design of this promising type of structure.

## Figures and Tables

**Table 1:** The table shows how the 9 radiating elements are excited in terms of power (in watts), amplitude (in volts), and phase (given as accumulated phase shift in degrees).

Element	Power [W]	Amplitude [V]	Phase [deg]
1	1	1.4142	0
2	0.8290	1.2876	127.37
3	0.6610	1.1498	254.74
4	0.5157	1.0155	382.11
5	0.3917	0.8851	509.48
6	0.2922	0.7645	636.85
7	0.2219	0.6661	764.22
8	0.1684	0.8504	891.59
9	0.1266	0.5032	1019.01

## Data Availability

Data are contained within the article.

## Abbreviations

The following abbreviations are used in this manuscript:

LWA	leaky-wave antenna
EM	electromagnetic
RMS	root mean square
